# Clinical effectiveness of posaconazole versus fluconazole as antifungal prophylaxis in hematology–oncology patients: a retrospective cohort study

**DOI:** 10.1002/cam4.225

**Published:** 2014-03-19

**Authors:** Hsiang-Chi Kung, Melissa D Johnson, Richard H Drew, Paramita Saha-Chaudhuri, John R Perfect

**Affiliations:** 1 Division of Infectious Diseases, Department of Internal Medicine, National Taiwan University HospitalTaipei, Taiwan; 2 Duke Clinical Research InstituteDurham, North Carolina; 3 Division of Infectious Diseases, Duke University Medical CenterDurham, North Carolina; 4 Campbell University College of Pharmacy and Health SciencesBuies Creek, North Carolina; 5 Department of Biostatistics and Bioinformatics, Duke University School of MedicineDurham, North Carolina

**Keywords:** Acute myeloid leukemia, fluconazole, fungal infections, myelodysplastic syndrome, posaconazole, prophylaxis

## Abstract

In preventing invasive fungal disease (IFD) in patients with acute myelogenous leukemia (AML) or myelodysplastic syndrome (MDS), clinical trials demonstrated efficacy of posaconazole over fluconazole and itraconazole. However, effectiveness of posaconazole has not been investigated in the United States in real-world setting outside the environment of controlled clinical trial. We performed a single-center, retrospective cohort study of 130 evaluable patients ≥18 years of age admitted to Duke University Hospital between 2004 and 2010 who received either posaconazole or fluconazole as prophylaxis during first induction or first reinduction chemotherapy for AML or MDS. The primary endpoint was possible, probable, or definite breakthrough IFD. Baseline characteristics were well balanced between groups, except that posaconazole recipients received reinduction chemotherapy and cytarabine more frequently. IFD occurred in 17/65 (27.0%) in the fluconazole group and in 6/65 (9.2%) in the posaconazole group (*P* = 0.012). Definite/probable IFDs occurred in 7 (10.8%) and 0 patients (0%), respectively (*P* = 0.0013). In multivariate analysis, fluconazole prophylaxis and duration of neutropenia were predictors of IFD. Mortality was similar between groups. This study demonstrates superior effectiveness of posaconazole over fluconazole as prophylaxis of IFD in AML and MDS patients. Such superiority did not translate to reductions in 100-day all-cause mortality.

## Introduction

Invasive fungal disease (IFD) is an important cause of morbidity and mortality in leukemic patients 1,2. Despite the advances of new antifungal agents and diagnostic tools, mortality from IFDs in these patients remains high 3. Strategies to address this problem include prophylactic, preemptive, and empirical administration of systemic antifungals 1,4. A randomized controlled clinical trial of antifungal prophylaxis with oral posaconazole reported overall mortality was reduced in patients with acute myeloid leukemia (AML) or myelodysplastic syndrome (MDS) undergoing induction chemotherapy relative to standard azole prophylaxis 5. In a second clinical trial in allogeneic stem cell transplant recipients with graft-versus-host disease (GVHD), posaconazole was as effective as fluconazole at preventing all IFDs and superior in preventing proven/probable invasive aspergillosis 6. As a result, primary prophylaxis with posaconazole has been recommended in select international guidelines for patients with malignancy at high-risk of IFDs 7–9.

The clinical effectiveness of posaconazole prophylaxis outside of clinical trials may vary depending on local epidemiology and clinical practice 10. Posaconazole was introduced at Duke University Hospital (DUH) in 2006. Before that time, most patients eligible for antifungal prophylaxis would be given fluconazole. Presently, there are no published studies evaluating the clinical effectiveness of posaconazole prophylaxis in the United States. To better understand the potential role of posaconazole in preventing IFD based on the local epidemiology, we aimed to determine the clinical effectiveness of posaconazole prophylaxis. The primary objective of this study was to compare the incidence of IFDs (including proven, probable, and possible cases) in select hematology/oncology patients who had received posaconazole prophylaxis compared to those who received fluconazole prophylaxis.

## Methods

This retrospective cohort study was conducted in compliance with the protocol, which was reviewed and approved by the Duke University Medical Center Investigational Review Board before any study related procedures were performed.

### Population

Study subjects included men and women ≥18 years of age admitted to DUH from July 1, 2004 to December 31, 2010 undergoing first induction or reinduction chemotherapy for AML or MDS and administered at least one dose of either posaconazole or fluconazole for IFD prophylaxis. Patients receiving other systemic antifungals, or diagnosed with IFD within the month prior to initiation of induction chemotherapy were excluded. No patient was included twice.

### Subject identification and data collection

AML or MDS patients (ICD9 codes: 208.0 acute leukemia, 206.8 acute leukemia, V10.6 leukemia, 205.0 acute leukemia, 208.9 unspecified leukemia, 238 MDS) who received chemotherapy (ICD9 code V58.1 or V58.11) at DUH between July 1, 2004 to December 31, 2010 were identified by query of an electronic medical record database. Subsequently, review of medical records was performed and data were recorded onto structured data abstraction forms. Data capture included demographics, underlying disease(s), description of chemotherapy, dates of neutropenia, length of stay, incidence and duration of fever, administration of antibacterials and antifungals, culture results from the DUH Clinical Microbiology Laboratory database, histopathology, galactomannan antigen from blood and bronchoalveolar lavage (BAL), chest computed tomography (CT) imaging studies, and survival. To ensure thorough and convenient documentation, a documentation platform based on Microsoft Access 2003 (Microsoft Corporation, Redmond, WA) was used.

### Treatment

Patients admitted to DUH for first induction or reinduction chemotherapy for AML or MDS received antifungal prophylaxis at the discretion of the oncology team, as there was no institutional protocol dictating prophylaxis during the study years. The attending physicians rotated inpatient clinical responsibilities weekly. Posaconazole suspension (Noxafil, Merck &Co, Inc. Whitehouse Station, NJ) was administered orally as 200 mg three times daily. Fluconazole was typically administered orally as 400 mg once daily (with adjustments based on renal function as necessary). Serum concentration monitoring was not routinely performed for patients receiving antifungal prophylaxis in our institution during the study period. Antibacterial prophylaxis was not routinely administered to AML/MDS patients in our institution during the study time frame, although antiviral prophylaxis with acyclovir was administered for those with a history of herpes simplex virus infection. No substantial changes in chemotherapy protocols were made during the study years.

### Definitions and endpoints

The primary endpoint was occurrence of an IFD (including proven, probable, and possible), as defined by the European Organization for Research and Treatment of Cancer (EORTC) and the Mycoses Study Group (MSG) 11. The secondary endpoint was death from any cause within 100 days after the first dose of induction chemotherapy. Other secondary endpoints included occurrence of fever, persistent fever unresponsive to broad-spectrum antibiotic treatment for ≥72 h, switch to other systemic antifungal therapy as empirical or preemptive therapy, pneumonia and lung infiltrates indicative of invasive fungal infections (dense, well-circumscribed lesions with or without a halo sign, air-crescent sign or cavity shown on CT). A positive galactomannan test was defined as two consecutive blood samples or a single BAL fluid sample with an index ≥0.5. Galactomannan was not evaluated when sampled on days with concomitant piperacillin/tazobactam treatment to avoid false positives. The observational period for determining all outcomes (except 100-day mortality) started at the first dose of chemotherapy and ended after stable recovery from neutropenia or at the time of discharge, whichever came first. Stable recovery from neutropenia was defined as a neutrophil count ≥500/mL for at least two consecutive days. All patients were followed up with regard to survival for 100 days after the start of chemotherapy.

### Data analysis

Subjects were considered evaluable as long as they received at least one dose of intended antifungal prophylaxis with posaconazole or fluconazole. Patients receiving at least one dose of posaconazole intended as prophylaxis between the start of chemotherapy and recovery from neutropenia were assigned to the posaconazole group.

The proportion of IFDs between posaconazole and fluconazole prophylaxis groups was compared using the chi-square test of association (or Fisher's exact test, as appropriate). The student's *t*-test was used for continuous variables. Survival at 100 days was also examined using Kaplan–Meier Survival Analysis. To assess predictors of IFD, select prespecified clinical data and prophylactic antifungal use were compared between those who experienced an IFD and those who did not, using univariate analysis followed by multivariable logistic regression. Variables with *P* < 0.1 on univariate analysis were considered for inclusion in the multivariable model. Multivariable logistic regression was performed using a full model, as well as forward selection and backwards elimination, retaining only variables with *P* < 0.05 in the final best-fit model. SAS (Version 9.3, SAS Institute, Cary, NC) was used for statistical analysis.

## Results

**Figure 1:**
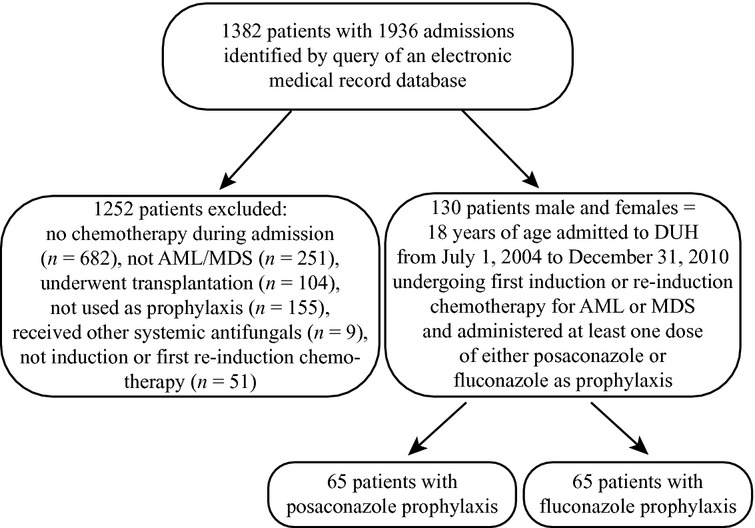
Flow chart of inclusion and exclusion of this study.

A total of 1382 patients were identified by query of an electronic medical record database. 1252 patients were excluded based on study inclusion/exclusion criteria; finally 130 patients were included (fluconazole, *n* = 65; posaconazole, *n* = 65; Fig. 1). Reasons for exclusion were as followed: no chemotherapy received during the admission (*n* = 682), not AML/MDS (*n* = 251), antifungal agent not used as prophylaxis (*n* = 155), no systemic antifungals (*n* = 9), and not induction or first reinduction chemotherapy (*n* = 51). For patients who received prophylaxis prior to 2006, fluconazole was used exclusively. However, only 34% of fluconazole subjects were from the years 2004–2006, and 66% were from 2007 to 2010. Even though posaconazole became available in 2007, it was only used in three study subjects that year, with the majority receiving it between 2008 and 2010. However, baseline characteristics (summarized in Table 1) were well-balanced between groups, with the exception of more patients receiving reinduction (*P* = 0.0077) and cytarabine (*P* = 0.026) in the posaconazole group.

**Table 1:** Characteristics of the study population by prophylaxis group

Characteristic	Fluconazole group (*n* = 65)	Posaconazole group (*n* = 65)	*P*-value
Age, years			
Mean + SD	60.2 + 14.4	58.9 + 14.7	0.63^1^
Median (range)	65 (22–83)	62 (21–82)	
Gender, number of patients (%)			
Male	42 (64.6)	43 (66.2)	0.85
Female	23 (35.4)	22 (33.9)	
Total cytarabine dosage during admission (mg), mean ± SD	680.1 ± 1007.9	1330.1 ± 2101.2	0.026
Duration of prophylaxis (days), mean ± SD	15.7 ± 8.9	18.2 ± 9.4	0.12
Duration of neutropenia (PMN <500/mm^2^, days), mean ± SD	24.2 ± 10.6	25.9 ± 11.6	0.36
Race, number of patients (%)			
Caucasian	54 (83.8)	52 (80.0)	
African–American	7 (10.8)	10 (15.4)	
Asian	1 (1.5)	2 (3.1)	
Native American	2 (3.1)	1 (1.5)	
Others	1 (1.5)	0 (0)	
First reinduction leukemia, no (%)	3 (4.6)	14 (21.6)	0.0077^2^
Secondary leukemia, no (%)	26 (40.0)	19 (29.2)	0.20
Chronic kidney disease, number of patients (%)	5 (7.7)	4 (6.2)	1.00^2^
Chronic obstructive pulmonary disease, number of patients (%)	3 (4.6)	1 (1.5)	0.62^2^
Diabetes mellitus, number of patients (%)	9 (13.9)	11 (16.9)	0.63

1 Student's *t*-test for independent samples (two-sided) for continuous variables.

2 Fishers's exact test (two-sided) for small numbers of categorical variable, others: chi-square test.

The incidence of invasive fungal infections (IFDs) and the causative pathogens are detailed in Table 2. Definite, probable, or possible IFDs occurred in 17/65 (27.0%) patients in the fluconazole group and in 6/65 (9.2%) in the posaconazole group (*P* = 0.012). Definite and probable IFDs occurred in seven (10.8%) and zero (0%), respectively (*P* = 0.013).

**Table 2:** IFD by prophylaxis group

IFD	Number of patients (%)		*P*-value
		
	Fluconazole group (*n* = 65)	Posaconazole group (*n* = 65)	
Patients with proven/probable IFDs	7 (10.8)	0 (0)	0.013
*Proven/Probable IFDs Pathogen*			
Mold	1 (1.5)		
Aspergillus	1 (1.5)		
Fusariosis	1 (1.5)		
Zygomycosis	1 (1.5)		
Yeast	1 (1.5)		
*Candida albicans*	1 (1.5)		
*C. glabrata*	1 (1.5)		
*C.guilliermondii*			
*C. krusei*			
Patients with possible IFDs	10 (15.4)	6 (9.2)	0.29
Total patients with IFDs	17 (27.0)	6 (9.2)	0.012

IFD, Invasive fungal disease.

**Figure 2:**
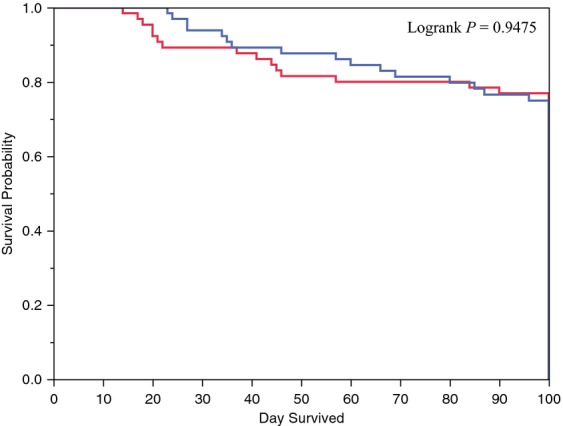
Overall survival (Kaplan–Meier plot). *Note*: Three cases were censored: all three were in the posaconazole group. Blue solid line: posaconazole prophylaxis; red dashed line: fluconazole prophylaxis.

No significant differences were observed for all-cause mortality at 100 days (23.1% fluconazole and 25.8% posaconazole; *P* = 0.72), respectively. Kaplan–Meier analysis of the time to death from any cause up to 100 days did not show survival differences within the two groups (*P* = 0.9475) (Fig. 2).

Other secondary endpoints are summarized in Table 3. Patients receiving posaconazole prophylaxis experienced fewer instances of persistent fever unresponsive to broad-spectrum antibiotic treatment for ≥72 h (27.7% and 61.5%; *P* = 0.0001). No significant differences were observed for incidence of fever (98.5% and 96.9%; *P* = 0.56), switch to other systemic antifungal therapy as empirical or preemptive therapy (43.1% and 56.9%; *P* = 0.11), or pneumonia and lung infiltrates indicative of invasive fungal infections shown on CT (15.4% and 23.1%; *P* = 0.27) in the posaconazole and fluconazole groups, respectively.

**Table 3:** Select clinical outcomes by prophylaxis group

Clinical outcomes	Fluconazole group (*n* = 65)	Posaconazole group (*n* = 65)	*P*-value
Persistent fever unresponsive to broad-spectrum antibiotic treatment for ≥72 h, no (%)	40 (61.5)	18 (27.7)	<0.0001
Fever, no (%)	63 (96.9)	64 (98.5)	0.56
Switch to other systemic antifungal therapy as empirical or preemptive therapy, number of patients (%)	37 (56.9)	28 (43.1)	0.12
Pneumonia and lung infiltrates indicative of invasive fungal infections shown on CT, no of patients (%)	15 (23.1)	10 (15.4)	0.27
ICU admission, no (%)	9 (13.9)	7 (10.8)	0.60
Inpatient length of stay days, mean ± SD	33.4 ± 19.7	33.3 ± 13.3	0.97
100-day mortality, no (%)	15 (23.1)	16 (25.8)^1^	0.72

1 Three subjects in the posaconazole group were lost to follow-up at 100 days. SD, standard deviation.

We assessed 17 prespecified variables that could be associated with our endpoint of IFD. The results of univariate and multivariate logistic regression analyses are shown in Tables 4 and 5, respectively. After forward and backward selection, two factors remained in the model. The global likelihood ratio *P*-value was <0.0001 (rejecting the null hypothesis that there was no relationship between any of the predictor variables and IFD). Fluconazole prophylaxis (vs. posaconazole prophylaxis, odd ratio [OR] 5.2, 95% confidence interval [CI] 1.6–15.4, *P* = 0.0053) and duration of neutropenia (OR 1.078, CI 1.033–1.125, *P* = 0.0005) were significantly associated with breakthrough IFD in our final model. Other variables such as concomitant bloodstream infection, total dose of corticosteroids, and secondary AML were not significantly associated with breakthrough IFD in our analysis.

**Table 4:** Univariate analysis of factors which may be associated with IFDs during antifungal prophylaxis

Factor	Patients with no IFD (*n* = 107)	Patients with IFD (*n* = 23)	*P*-value
Posaconazole prophylaxis, no (%)	59 (55.1)	6 (26.9%)	0.012
Duration of neutropenia (days), mean ± SD	23.5 ± 9.6	32.4 ± 14.3	0.0003
Concomitant bloodstream infection, no (%)	57 (53.2)	18 (34.8)	0.028
Total dose of steroid (mg)	80.8 ± 37.9	98.6 ± 32.7	0.038
Intensive care unit admission, no (%)	8 (7.5)	8 (34.8)	0.0015
Secondary AML, no (%)	33 (30.8)	12 (52.2)	0.051
Receipt of total parenteral nutrition during hospitalization, no (%)	2 (1.9)	2 (8.7)	0.14
Chronic obstructive pulmonary disease, no (%)	2 (1.9)	2 (8.7)	0.14
Relapse, no (%)	15 (14.0)	2 (8.7)	0.74
Prior fungal colonization, no (%)	5 (4.7)	2 (8.7)	0.61
Presence of central lines, no (%)	89 (83.2)	21 (91.3)	0.53
Receipt of corticosteroids during hospitalization, no (%)	102 (95.3)	23 (100)	0.59
Age, mean ± SD	59.5 ± 14.5	59.9 ± 14.8	0.90
Mucositis during hospitalization, no (%)	21 (19.6)	1 (4.4)	0.12
Baseline CrCl <30 mL/min, no (%)	9 (8.4)	0 (0)	0.36
Acute renal failure (CrCl < 10 mL/min) during hospitalization, no (%)	7 (6.5)	2 (8.7)	0.66
Diabetes mellitus, no (%)	18 (16.8)	2 (8.7)	0.53
Male gender, no (%)	70 (65.4)	15 (65.2)	0.99
Total cytarabine received during this admission (mg), mean ± SD	966.9 ± 1644.3	1183.1 ± 1832.2	0.58

IFD, possible, probable or definite invasive fungal disease; Cr Cl, creatinine clearance.

**Table 5:** Factors associated with breakthrough IFD by multivariate logistic regression

Variable	Odds ratio	95% CI	*P*-value
Fluconazole prophylaxis	4.975	1.610, 15.385	0.0053
Neutropenia	1.078	1.033, 1.125	0.0005

95% CI, 95% confidence interval; BSI, bloodstream infection.

## Discussion

Previous published studies have prospectively evaluated the epidemiology of IFDs in AML patients undergoing first remission-induction chemotherapy before and after the introduction of posaconazole prophylaxis as a standard of care. When comparing 82 patients who received topical polyene prophylaxis with 77 patients who received posaconazole prophylaxis, the number of febrile days, the incidence rate of IFDs and aspergillosis and the duration of hospitalization decreased significantly among those patients receiving posaconazole 12. Another single-center retrospective study assessed all patients with AML/MDS receiving induction chemotherapy and all patients with GVHD treated from 2006 to 2008 13. Overall IFD rates were reduced from 47% (non-posaconazole prophylaxis group, 56 patients) to 35% (posaconazole prophylaxis group, 34 patients), but statistical significance was not reported by these authors. Initiation of antifungal therapy was significantly less frequent and mortality was higher (15% vs. 7%) in the posaconazole prophylaxis group. Such outcomes might be explained (in part) by the presence of more patients with severe GVHD in the posaconazole prophylaxis group.

In this study, we determined the clinical effectiveness of posaconazole in preventing IFD in our institution. Patients in both the posaconazole and fluconazole groups were well-balanced in all assessed parameters except there were more patients undergoing reinduction chemotherapy and higher total doses of cytarabine administered in the posaconazole group. Receipt of high-dose cytarabine is an established risk factor for IFDs 14. Despite the greater potential for increased IFD risks in those receiving posaconazole with more reinduction chemotherapy and higher doses of cytarabine, a significant decrease in IFDs was observed in this group compared with the fluconazole group. This confirms findings of a large randomized trial 5, prospective cohort 12,15, and retrospective cohort 13 in Europe that posaconazole is more effective in the prophylaxis of IFDs than fluconazole.

In addition to the reduction in IFDs, our study also demonstrated that patients receiving posaconazole prophylaxis had fewer persistent fevers unresponsive to broad-spectrum antibiotic treatment for ≥72 h. In contrast, we did not find significant differences in duration of hospitalization, shifting to other empirical or preemptive antifungal therapy (43.1% in posaconazole group and 56.9% in fluconazole group; *P* = 0.12) or all-cause mortality.

Clearly, our study in this “real-world” setting has several limitations. Our observation period for IFDs was relatively limited (to minimize bias from other intervening factors during long-term followup), although subjects were followed up for mortality for up to 100 days. In addition, diagnostic uncertainty exists in the determination of IFD, even in prospective evaluations. As the study was retrospective in nature, certain data were left undocumented. As there was no preexisting institutional protocol dictating antifungal prophylaxis or diagnostic work-up of IFD in this population during the years studied, assignment to treatments and performance of diagnostic procedures were based on individual clinician's judgment. Due to potential harmful effects of invasive procedures, these aggressive diagnostic tests would not typically be performed while a patient is at the nadir of their blood counts. Commonly, this leads to both diagnostic delays and uncertainties in this clinical setting. Therefore, the overall incidence of IFDs could potentially be underestimated in our dataset. In a recent prospective Italian study which a predefined diagnostic strategy was implemented, the incidence of proven and probable IFDs was higher than our assessment of IFDs with 23.2% in those receiving posaconazole 15. In contrast, a prospective German cohort reported the incidence of breakthrough IFDs to be only 3.9% in those receiving posaconazole 12. Possible underestimation of disease, however, should affect both treatment groups equally and hence would not introduce bias.

Furthermore, the retrospective nature of this study and complexities of the patient population make determination of attributable mortality or causal-adverse events quite difficult. Thus, only all-cause mortality was assessed.

Understanding the local epidemiology of IFDs in hematology patients provides insight to future study design and is a useful reference for determining optimal clinical practice in our hospital and all large cancer centers. As shown in this retrospective study using real-world data, clearly fewer IFDs are observed with the broader spectrum posaconazole prophylaxis and confirm the controlled randomized study 5. These results occurred despite no significant attention to posaconazole bioavailability, which can be challenging in this patient population. Although in this single-center study we did not show a statistical difference in overall mortality at 100 days, surely the ability to avoid IFDs is likely to have a direct clinical benefit and might be missed because of the small size of our study or the impact of underlying at 100 days. This study also supports the cost-effectiveness of an antifungal prophylaxis strategy which is likely to be favorable.

## Conclusion

We observed superior effectiveness of posaconazole to fluconazole for the prophylaxis of IFD in AML and MDS patients. In addition to the prophylactic agent used, the duration of neutropenia also impacts the incidence of breakthrough IFD. Despite a decreased incidence in IFDs in the posaconazole group, no reductions in 100-day all-cause mortality were observed.
